# A novel peptide inhibitor of TRPM2 channels improves recovery of memory function following traumatic brain injury

**DOI:** 10.3389/fnsyn.2025.1534379

**Published:** 2025-04-25

**Authors:** James E. Orfila, Robert M. Dietz, Christian Schroeder, Olivia P. Patsos, Amelia Burch, Kiara E. Bahamonde, Kelley A. Coakley, Danelle J. Carter, Amy C. Clevenger, Tara B. Hendry-Hofer, Tuan D. Le, Joseph K. Maddry, Steven G. Schauer, Vikhyat S. Bebarta, Paco S. Herson

**Affiliations:** ^1^Department of Neurological Surgery, The Ohio State University College of Medicine, Columbus, OH, United States; ^2^Department of Pediatrics, University of Colorado School of Medicine, Aurora, CO, United States; ^3^Department of Anesthesiology, University of Colorado School of Medicine, Aurora, CO, United States; ^4^Department of Emergency Medicine, University of Colorado School of Medicine, Aurora, CO, United States; ^5^Department of Epidemiology and Biostatistics, The University of Texas Tyler School of Medicine, Tyler, TX, United States; ^6^CU Center for COMBAT Research, University of Colorado School of Medicine, Aurora, CO, United States; ^7^Uniformed Services University of the Health Sciences, Bethesda, MD, United States; ^8^Brooke Army Medical Center, San Antonio, TX, United States; ^9^59th Medical Wing, San Antonio, TX, United States

**Keywords:** traumatic brain injury, neuroprotection, long-term potentiation, cognition, histology

## Abstract

Traumatic Brain Injury (TBI) is a leading cause of mortality and morbidity in adults and can lead to long-term disability, including cognitive and motor deficits. Despite advances in research, there are currently no pharmacological interventions to improve outcomes after TBI. Studies suggest that non-selective transient receptor potential melastatin 2 (TRPM2) channels contribute to brain injury in models of ischemia, however TRPM2 remains understudied following TBI. Thus, we utilized TRPM2 KO mice and a novel TRPM2 inhibiting peptide, tatM2NX, to assess the role of TRPM2 in TBI-induced injury and functional recovery. This study used histological analysis of injury, neurobehavior and electrophysiology to assess the role of TRPM2 on injury and cognitive recovery (memory) impairments using the controlled cortical impact (CCI) model to induce TBI in mice. Histological analysis used to investigate brain injury volume at 7 days after TBI showed sex differences in response to injury in TRPM2 KO mice but no pharmacological effects in our WT mice. A contextual fear-conditioning task was used to study memory function 7 or 30 days after TBI and demonstrates that sham-operated mice exhibited significant freezing behavior compared to TBI-operated mice, indicating impaired memory function. Mice administered tat-M2NX 2 h after TBI exhibited a significant reduction of freezing behavior compared to control tat-scrambled (tat-SCR)-treated mice, suggesting improvement in memory function after TBI. To test the effect of TBI on hippocampal long-term potentiation (LTP), a well-established cellular model of synaptic plasticity associated with changes in learning and memory, extracellular field recordings of CA1 neurons were performed in hippocampal slices prepared 7 days after TBI. Consistent with our behavioral testing, we observed impaired hippocampal LTP in mice following TBI (tat-SCR), compared to sham control mice. However, mice treated with tat-M2NX after TBI exhibited preserved LTP, consistent with the improved memory function observed in our behavioral studies. While this data implicates TRPM2 in brain pathology following TBI, the improvement in memory function without providing histological protection suggests that administration of tatM2NX at an acute time point differentially affects hippocampal regions compared to cortical regions.

## Introduction

It is estimated that more than 1.5 million Americans suffer a traumatic brain injury (TBI) annually, defined as a blunt or penetrating injury to the head that alters brain function (Gardner and Zafonte, 2016). Importantly, TBI is also a major health issue in the U.S. military, with estimates of TBI prevalence as high as 23% of returning service members (Lindquist et al., 2017; Le et al., 2023). TBI is often associated with long-term disabilities, predominantly a reduction in working memory, difficulties learning new information, and decreased executive function and reasoning. Unfortunately, targeted therapies to improve survival and long-term outcomes following TBI are currently limited. Clinical trials in the civilian population have shown that neuroprotective strategies (compounds known to reduce histological injury) are extremely difficult to translate to improved outcomes (Mallah et al., 2020; Wright et al., 2014; Zoerle et al., 2017). The mechanisms of acute brain injury and progression to chronic dysfunction are complex and understudied. Emerging evidence indicates that injury following TBI has multiple phases, with early brain injury (primary) activating a complex cascade of molecular and cellular processes such as oxidative stress & neuroinflammation (secondary) that contributes to long term functional dysfunction. Consistent with the lack of translation, emerging data indicates that magnitude of primary histological injury does not directly correlate with functional recovery (Mallah et al., 2020). Therefore, therapeutic strategies that target secondary injury and long-term functional recovery are critically important for the advancement of therapies from the bench to the bedside. The current study uses the well-established controlled-cortical impact (CCI) model of moderate/severe TBI in combination with behavioral and cellular models of learning and memory to assess subacute and chronic outcomes following TBI (Clevenger et al., 2018). Further, we assessed the role of the oxidative-stress sensitive ion channel, transient receptor potential melastatin 2 (TRPM2), in TBI-induced primary and secondary injury.

The role of TRPM2 channels have been well studied in cellular models of oxidative stress and animal models of ischemia–reperfusion. TRPM2 channels are expressed in neurons throughout the brain, including the hippocampus, striatum, cortex and cerebellum and has been implicated in non-ischemic pathologies such as Alzheimer’s disease, Parkinson’s disease, dementia and bipolar disorder (Belrose and Jackson, 2018; Rather et al., 2023). We have extensive evidence for a sexually dimorphic response of TRPM2-induced acute injury following global and focal cerebral ischemia. We have demonstrated that acute pharmacological inhibition or genetic knockout/knockdown of TRPM2 channels reduces neuronal injury (neuroprotection) only in males, with no effect on acute injury observed in females (Jia et al., 2011; Nakayama et al., 2013; Shimizu et al., 2016). Interestingly, we recently demonstrated that delayed inhibition of TRPM2 channels following global cerebral ischemia enhanced cognitive recovery in both male and female animals, indicating a chronic activity of TRPM2 channels that contributes to secondary injury in both sexes, as indicated by improved neurobehavioral memory function and enhanced hippocampal synaptic plasticity (Dietz et al., 2020). However, the role of TRPM2 channels following TBI has been relatively understudied. There have been reports of increased TRPM2 expression weeks after TBI (Cook et al., 2010), implicating these channels in chronic secondary injury. Here, we use the TRPM2 knockout mouse and our novel TRPM2 channel antagonist (tat-M2NX) to assess the role of TRPM2 channels in acute and secondary injury. We hypothesize that TRPM2 is activated acutely in male brain and contributes to acute histological injury specifically in males and that TRPM2 is persistently activated in the hippocampus of both male and female mice, leading to reduced memory and impairments of hippocampal synaptic plasticity.

## Methods

### Experimental animals

All experimental protocols were approved by the Institutional Animal Care and Use Committee and conformed to the National Institutes of Health guidelines for the care and use of animals in research. Adult (2–4 month) male and female C57Bl/6 or TRPM2 KO mice were blindly randomized to experimental or control groups, minimizing subjective bias and controlling for sex effects of TBI. Our translational animal experiments were conducted in accordance with the ARRIVE guidelines (Kilkenny et al., 2011; Percie du Sert et al., 2020). All mice were housed in a standard 12 h light–dark cycle with access to food and water ad libitum. Investigators administrating drug were double blinded by not knowing the animal treatment and therapeutic being administered. All analyses were performed by observers blinded to treatment allocation. All methods utilized in this study can be found in our previous publications (Clevenger et al., 2018; Dietz et al., 2020; Orfila et al., 2018; Orfila et al., 2019).

A total of 197 mice were used for the current study. A total of 5 male KO, 3 female KO, 17 WT male and 0 WT female mice were excluded as they died prior to pre-determined experimental endpoint. No differences in survival rate was observed between experimental groups and overall survival rate was greater than 85%.

### Traumatic brain injury

Anesthesia was induced using 4% inhaled isoflurane, followed by maintenance with 1.5–2.5% inhaled isoflurane. After anesthetic induction, a 4 mm circular right craniotomy was completed using a micromotor drill (Stoelting, Wood Dale, IL) aligned 2.5 mm anterior to the lambdoid suture and 2.7 mm lateral to the sagittal suture using a stereotactic setup. Following exposure of the dura mater, a 3 mm flat-tipped impact device (Impact One Stereotaxic Electromagnetic Controlled Cortical Impactor (CCI), Leica Biosystems, Buffalo Grove, IL) was used to deliver a single, rapid impact using the following standardized parameters: angle 10° to the right of vertical, speed 3 ± 0.02 m/s, duration 500 msec. The depth of impact varied from 0 mm for sham and 2 mm for moderate injury. Following impact, hemostasis was achieved, the bone flap was replaced, and the skin closed. Each animal was given 1,000 μL (sc) normal saline (NS) at the end of surgery. When each animal demonstrated adequate respiratory effort, it returned to its cage to continue recovery.

### Quantification of injury volume

For quantification of injury volume, one set of serial tissue sections (6 μm thickness at 100 μm intervals) stained with hematoxylin and eosin (H&E) was analyzed using quantitative stereologic analysis to quantify injury volume according to the Cavalieri principle, as described previously by our group (Clevenger et al., 2018). A Leica photomicroscope (Leica Microsystems, Buffalo Grove, IL) and a StereoInvestigator program (Stereologer 2000, SRC, Tampa, FL) were used for this purpose. For each animal, a total of 8 standardized sections were analyzed to calculate the total injury volume in cubic millimeters. Injured area was traced at 1.25x magnification and contralateral cortical tracing used to adjust for missing tissue, and tissue depth was calculated at 40x magnification.

### Hippocampal slice preparation

Hippocampal slices were prepared from young adult male C57Bl/6 mice at either 7 or 30 days after recovery from TBI. Animals were anesthetized with 3% isoflurane in an O_2_ enriched chamber. Mice were decapitated, and the brains quickly extracted and placed in ice-cold (2–5°C) oxygenated (95% O_2_/5% CO_2_) artificial cerebral spinal fluid (aCSF – pH 7.4 ± 0.05) composed of the following (in mmol/L): 125 NaCl, 2.5 KCl, 25 NaHCO_3_, 1.25 NaH_2_PO_4_, 2 CaCl_2_, 1 MgCl_2_, 12 glucose. Horizontal hippocampal slices (300–350 μm thick) were cut with a Vibratome 1000 (Leica) and transferred to a holding chamber containing aCSF at room temperature for 1.5–2 h before recording.

A total of 49 mice were used for hippocampal field potential recordings. For details of number of mice used per experiment, refer to Supplementary materials.

### Electrophysiology

For synaptically evoked potentials, hippocampal slices were placed on a temperature controlled (32 ± 1°C) interface chamber perfused with oxygenated aCSF at a rate of 1.5 mL/min. Extracellular field recordings were performed by stimulating the CA3 Schaffer collaterals and recording the field excitatory postsynaptic potential (fEPSP) in the stratum radiatum of the CA1 (CA3-CA1 axis). fEPSPs were adjusted to 50% of the maximum slope and test pulses were evoked at a rate of 0.05 Hz. A 20 min stable baseline period was established before delivering a theta burst stimulation (TBS) train (4 pulses delivered at 100 Hz in 30 ms bursts, repeated ten times with 200 ms inter-burst intervals). Following TBS, the fEPSP was recorded for 60 min. Analog fEPSPs were amplified (1000×) and filtered through a pre-amplifier (Grass Instruments, Model P511) at 0.03 Hz–1.0 kHz, digitized at 1.0 kHz and stored on a computer for later off-line analyses (Clampfit 10.4, Axon Instruments). The derivative (dV/dT) of the initial 2–3 ms onset of the fEPSP slope was measured, and the amount of potentiation calculated as the percent change from baseline (the averaged 10 min slope value from 50 to 60 min post-TBS divided by the averaged slope value 10 min prior to TBS). For the time course graphs, normalized fEPSP slope values were averaged and plotted as the percent change from baseline and referenced to 100%.

### Contextual fear conditioning

The contextual fear conditioning paradigm was utilized as a hippocampal-dependent memory task (Dietz et al., 2020; Rudy and O'Reilly, 2001). The apparatus consisted of two fear conditioning chambers with shock grid floors, consisting of 16 stainless steel rods connected to a shock generator (Colbourn Instruments, Model H13-15, Whitehall, PA, USA). Mice were transported in white buckets during the training and testing sessions. During training on day 7 or 30 days after TBI, mice were allowed to habituate in the conditioning chamber for 2-min and returned to its home cage. 20–30 min following habituation, mice were again placed in the chamber for 2 minutes, pre-exposure sessions followed by a foot shock (2-s/1.0 mA electric shock) immediately after the second exposure. Following the shock, mice were immediately returned to their home cages. Memory was tested 24 h later by transporting mice in white buckets and placed into the same chambers to elicit associative memory. Memory was determined by percentage of freezing behavior, measured in 10-s intervals across a 5-min test by a blinded observer and was defined as the absence of movement except for heart beat/respiration.

### Tissue isolation and preparation

Following behavioral testing (7 days post-injury), animals were transcardially perfused under isoflurane anesthesia with phosphate-buffered saline (0.1 M, pH 7.4) for 5 min, followed by 5 min of fixation with freshly prepared 4% paraformaldehyde (PFA). Their brains were removed, allowed to post-fix in 4% PFA for 24 h, and embedded in paraffin, as previously described (Clevenger et al., 2018). Coronal sections were cut in 6 μm thickness, and every sixth section was mounted in series onto slides for further processing. The interval between serial slide groups was 100 μm.

### Statistical analysis

Analysis performed by separate investigator from individual performing the experiment in a blinded manner. All data are continuous and presented as mean ± SEM. Normality was confirmed for all groups using Shapiro–Wilk test. Power analysis prior to study was performed using G*Power software. Based upon precious results, to observe a 40% change in LTP between two groups with a standard deviation of 15 and an alpha error of 5% and a beta error of 80%, a group of 4 slices per group are required. In consideration of biologic variability, no more than 2 slices per animal used in analysis per condition. For behavior studies, to observe a 30% change in freezing behavior between two groups with a standard deviation of 15%, with an alpha error of 5% and a beta error of 80%, 8 animals per group are require for behavior experiments. Statistical analysis of all data was determined using the one-way analysis of variance (ANOVA) and *post-hoc* Dunnett’s test for comparison of multiple groups compared against the control group. Differences were considered statistically significant with *p* < 0.05, as indicated by the asterisks.

## Results

### TRPM2 KO reduces TBI-induced injury specifically in males

**Figure 1:**
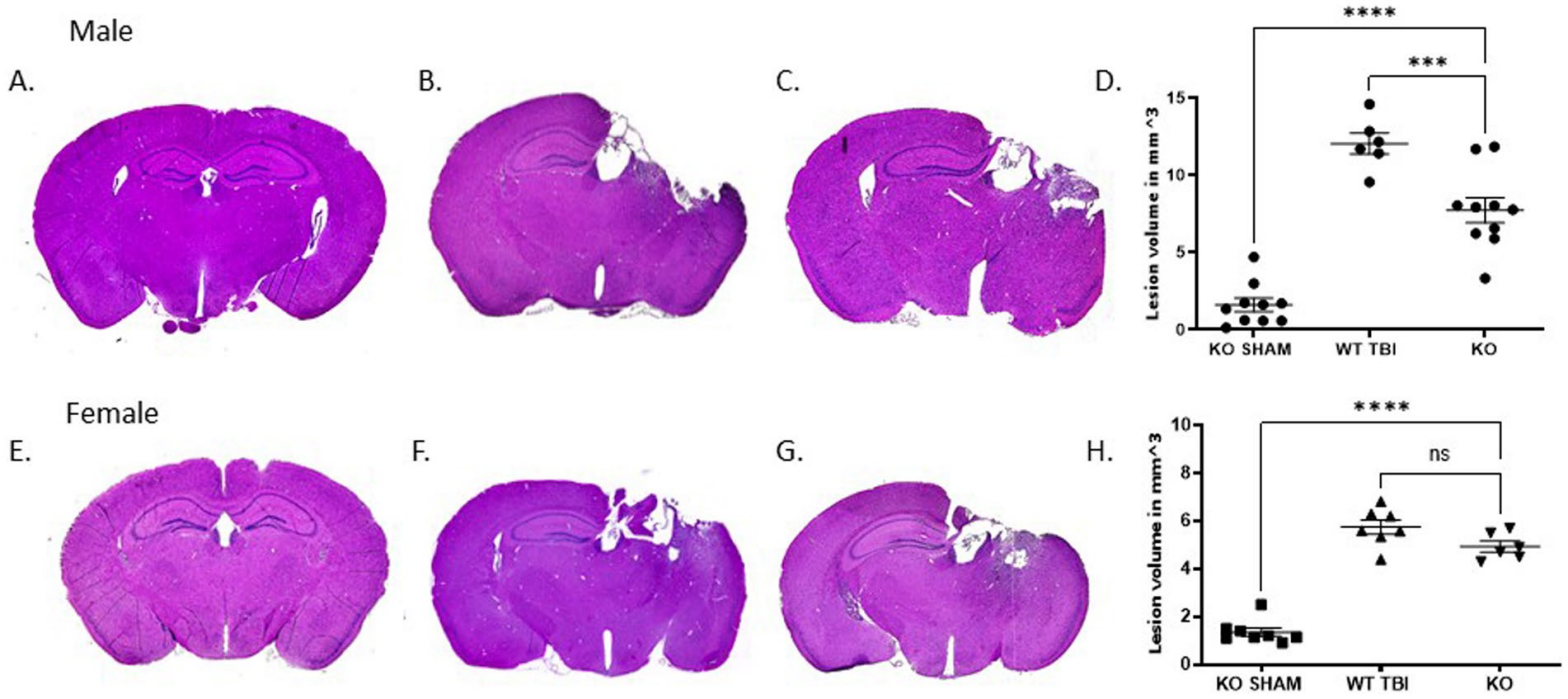
TRPM2 KO confirmed neuroanatomical protective effects from TBI specifically in male mice. Stereologic quantitative analysis of injury volume at 7 days after TBI demonstrates that a 2 mm impact depth in TRPM2 -/- was significantly reduced when compared to female mice. **(A–C)** Gross examination of lesion volume in male KO sham, WT TBI and KO TBI mice brains 7 days after TBI when compared to KO TBI mice. **(D)** Summary bar graph in KO Sham (1.6 ± 1.4 mm^3^; *n* = 10), WT TBI (12.1 ± 1.7 mm^3^; *n* = 6) and KO (7.7 ± 2.6 mm^3^; *n* = 10). **(E–G)** Gross examination of lesion volume in female KO sham and WT TBI mice brains 7 days after TBI when compared to KO TBI mice. **(H)** Summary bar graph in female KO Sham (1.4 ± 0.5 mm^3^; *n* = 8), WT TBI (5.8 ± 0.8 mm^3^; *n* = 7) and KO (4.9 ± 0.6 mm^3^; *n* = 6). Statistical analysis using 1-way ANOVA (*n* = 6–10 mice per group; *p* < 0.05, 1-Way ANOVA). Error bars indicate standard error of the mean.

To determine if TRPM2 channels contribute to injury volume following traumatic brain injury, stereological analysis was done 7 days after TBI in both male and female TRPM2 KO mice. Figures 1A–C are representative tissue sections stained with H&E from each animal group. As described previously, CCI produced visible injury in the cortex, hippocampus and corpus collosum (Clevenger et al., 2018). In male sham TRPM2 KO mice (Figures 1A,D), a small cortical injury is associated with the craniectomy (1.6 ± 1.4 mm^3^; *n* = 10). Male TRPM2 KO injured mice (Figures 1C,D) showed a significant decrease in injury volume (7.7 ± 2.6 mm^3^; *n* = 10) when compared to WT TBI-injured mice (12.1 ± 1.7 mm^3^; *n* = 6, *p* < 0.05, 1 way ANOVA, Figures 1B,D). Female WT TBI-injured mice sham operated mice (Figures 1E,H) showed a small cortical injury associated with the craniectomy (1.4 ± 0.5 mm^3^; *n* = 8). Female KO injured mice (Figures 1F,H) showed a significant injury when compared to sham operated mice (4.9 ± 0.6 mm^3^; *n* = 6; *p* < 0.05, 1-Way ANOVA). However, female TRPM2 KO mice (Figures 1G,H) did not exhibit reduced injury magnitude compared to WT TBI mice (5.8 ± 0.8 mm^3^; *n* = 7). These data indicate sex differences in response to injury, consisted with our previous studies of ischemia–reperfusion injury (Jia et al., 2011; Nakayama et al., 2013; Shimizu et al., 2016; Shimizu et al., 2013).

### Acute inhibition of TRPM2 channels fails to reduce injury following TBI

**Figure 2:**
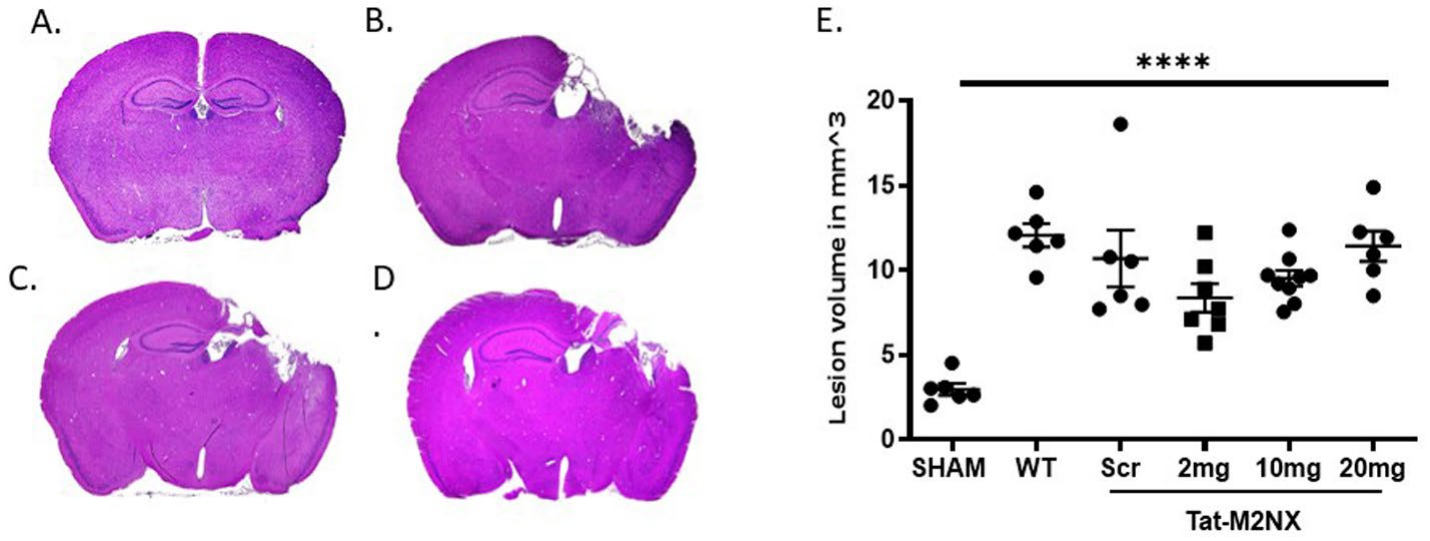
Acute tatM2NX administration fails to reduce injury. Stereologic, quantitative analysis of injury volume. Gross examination of the brain 7 days after TBI demonstrates that a dose response of tat-M2NX did not significantly decrease brain injury lesion volume. **(A)** sham mice displayed significantly smaller injury volume (2.9 ± 0.3 mm^3^; *n* = 6; *p* < 0.05) when compared to **(B)** male WT TBI mice (12.06 ± 1.663, *n* = 6), **(C)** Tat-Scr treated mice (10.7 ± 4.1 mm^3^; *n* = 6) and **(D)** tat-M2NX 10 mg/kg treated (9.525 ± 1.418, *n* = 9). **(E)** Summary bar graph across all groups demonstrates statistically significant injury volume in mice treated with tat-M2NX at 2, 10 and 20 mg/kg (8.4 ± 2.2 mm^3^, *n* = 7 and 9.5 ± 1.4 mm3, *n* = 9; 11.4 ± 2.2 mm3 *n* = 6; respectively) comparison to sham, *n* = 5–9 animals per group, analyzed by 1-way ANOVA. (WT TBI group same as used in Figure 1). Error bars indicate standard error of the mean.

As a result of the observation of male-specific reduction of injury in TRPM2 KO mice, we focused on the effect of acute administration of tat-M2NX in male animals. To determine whether inhibiting TRPM2 channels with our novel peptide, tat-M2NX, would decrease cortical injury volume, mice were administered tat-M2NX intravenously at varying doses (2, 10 or 20 mg/kg) 2 h after injury to determine its protective effect. Dose range chosen based upon previous studies that have demonstrated maximal effect at 20 mg/kg tatM2NX in models of cerebral ischemia (Shimizu et al., 2016; Dietz et al., 2020). Stereological analysis of histological injury performed 7 days after recovery from TBI. Figures 2A,D shows that sham operated mice displayed a small cortical injury associated with the craniectomy (3.0 ± 0.3 mm^3^; *n* = 6). Male WT TBI mice (Figures 2B,D) showed a significant increase in injury volume (12.1 ± 1.7 mm^3^, *n* = 6, WT – TBI) group same as used in Figure 1, when compared to sham mice (*p* < 0.05, 1-Way ANOVA). Figures 2D,E shows tat-M2NX administered at 2, 10 or 20 mg/kg (8.4 ± 2.2 mm^3^, *n* = 7 and 9.5 ± 1.4 mm^3^, *n* = 9; 11.4 ± 2.2 mm^3^ *n* = 6; respectively) did not significantly decrease injury volume when compared to control tat-scrambled (tat-SCR – scrambled molecule that contains all the same amino acids as the M2NX peptide in a new random order) TBI treated mice (10.7 ± 4.1 mm^3^; *n* = 6, Figures 2C,E). These data confirm previous studies demonstrating lack of functional activity of tatSCR (Shimizu et al., 2016; Cruz-Torres et al., 2020). These data suggest that pharmacologically inhibition of TRPM2 channels fail to provide histological protection.

### TRPM2 KO preserves synaptic plasticity following TBI

**Figure 3:**
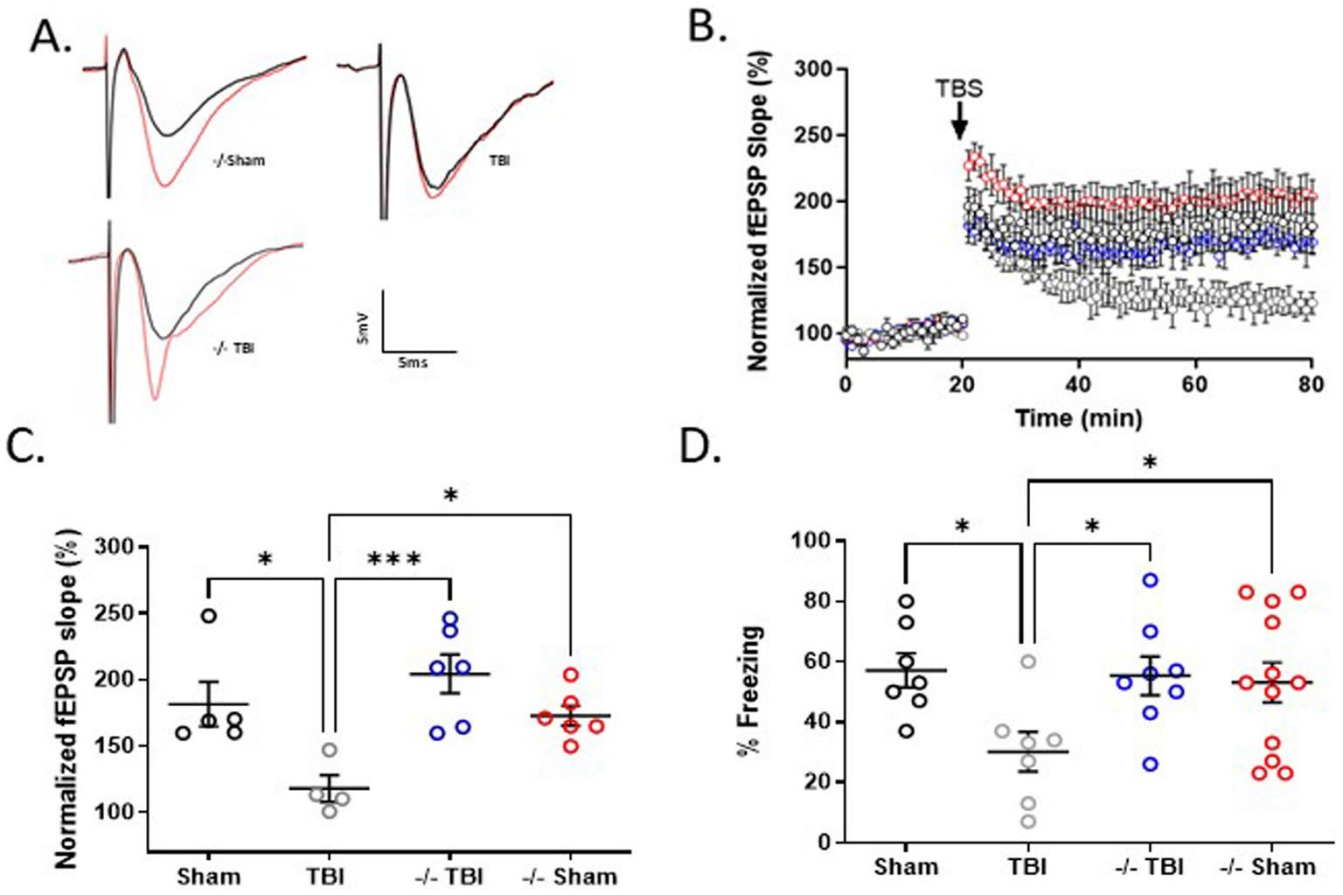
TRPM2 KO mice exhibit enhanced hippocampal plasticity and memory function 7 days after recovery from TBI. **(A)** Example fEPSPs from sham, TBI and −/-TBI operated mice at 7 days after injury before (black) and after (red) TBS in sch-CA1 hippocampal pathway. **(B)** Time-course of fEPSP slope from the hippocampus in sham mice (181.5 ± 16.82; *n* = 5; black filled circles), TBI (117.9 ± 10.09, *n* = 4; gray circles) and −/− mice in (204.3 ± 14.64, *n* = 6; blue circles) and −/− sham (172.8 ± 7.542, *n* = 6; red circles) 7 days after TBI. Arrow indicates timing of TBS (40 pulses). **(C)** Quantification of change in fEPSP slope after 60 min following TBS normalized to baseline, set at 100%. Each point represents a hippocampal slice that was recorded with no more than two slices per animal used. **(D)** Memory impairment 7 days after TBI. Adult mice displayed memory dysfunction after contextual fear conditioning. Quantification of freezing behavior 24 h after contextual fear conditioning in a novel environment (*n* = 7–12/group. *p < 0.05 compared to tat-scrambled, 1-Way ANOVA) behavior was scored.

To assess the role of TRPM2 channels on hippocampal function, we performed electrophysiology experiments to quantify hippocampal synaptic plasticity (LTP) in male TRPM2 KO mice 7 days after recovery from TBI. Electrophysiologic data was collected using extracellular field recording (Figure 3A) in the CA1 area of acute hippocampal slices 7 days following TBI and compared to slices collected from sham-operated control mice in both WT and TRPM2 KO mice. In control slices, a brief TBS (40 pulse TBS, 100 Hz) resulted in LTP of 181.5 ± 16.8% (*n* = 5). Figures 3B,C shows a significant impairment of LTP in WT mice (117.9 ± 10.1%; *n* = 4) 7 days after TBI when compared to sham controls (*p* < 0.05, 1-Way ANOVA). In contrast, TBI did not alter LTP in TRPM2 KO mice 7 days after TBI compared to TRPM2 Sham mice (204.3 ± 14.6%; *n* = 6 and 172.8 ± 7.5; *n* = 6, respectively). There were no differences in paired pulse ratio or input–output characteristics in any of the conditions studied (Supplementary Table 1). These data suggest that male TRPM2 KO mice displayed a preservation of hippocampal synaptic function when compared to WT TBI mice.

To extend our observation of TRPM2 KO preventing TBI-induced deficits in LTP, we performed contextual fear conditioning to measure memory function in TRPM2 KO mice 7 days after recovery from TBI. Figure 3D shows that WT sham operated mice exhibited intact short-term memory, as evidence by freezing behavior (57.1 ± 5.7%; *n* = 7). WT males tested 7 days after TBI demonstrated reduced freezing behavior (30.1 ± 6.6%; *n* = 7), indicating impaired memory function. In contrast, male TRPM2 KO mice tested 7 days after TBI exhibited intact memory function (55.3 ± 6.6%; *n* = 8), comparable to sham TRPM2 KO levels (53.1 ± 6.6%; *n* = 12). A one-way ANOVA revealed wild-type TBI mice exhibited reduced freezing when compared to WT sham, sham TRPM2 KO and TRPM2 KO (*p* < 0.05, 1-Way ANOVA), indicating reduce memory in WT mice following TBI that was not observed in TRPM2 KO mice.

### Acute inhibition of TRPM2 channels preserves synaptic plasticity and memory function following TBI

**Figure 4:**
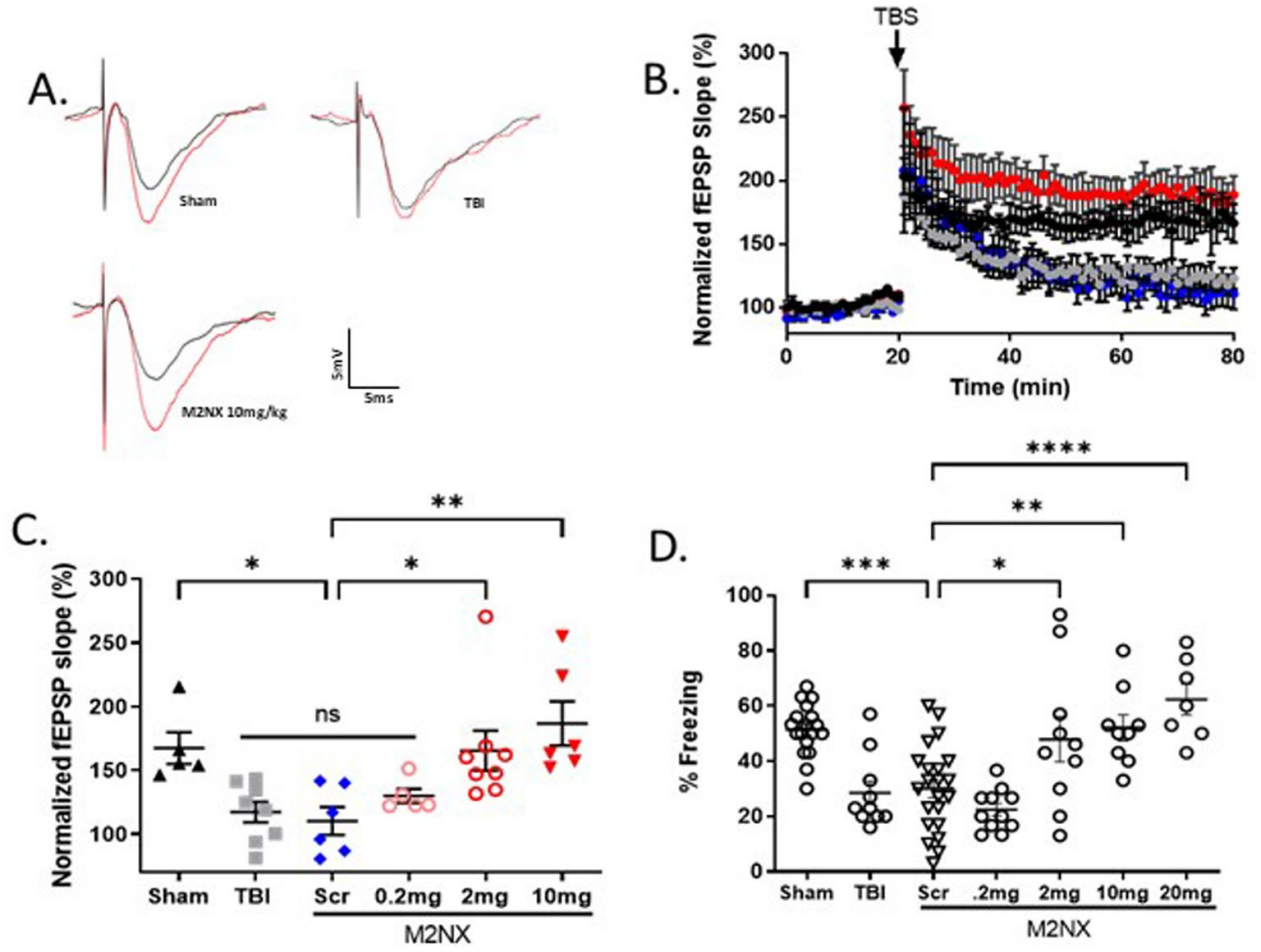
Acute taM2NX administration enhances hippocampal plasticity and memory function 7 days after recovery from TBI. **(A)** Example fEPSPs from sham-operated control mice at 7 days after injury before (black) and after (red) TBS in sch-CA1 hippocampal pathway. **(B)** Time-course of fEPSP slope from the hippocampus in sham mice (167.5 ± 12.43; *n* = 5; black triangle), TBI (117.3 ± 8.180, *n* = 8; gray filled squares), Scr (110.4 ± 10.9%; *n* = 6, blue diamonds), tat-M2NX 0.2 mg/kg (129.9 ± 5.6%, *n* = 5, rose circles), tat-M2NX 2.0 mg/kg (165.5 ± 15.7% *n* = 8, red circles) and tat-M2NX 10 mg/kg (186 ± 17.28, *n* = 6; red filed triangle). Treatment with tat-M2NX at 2.0 or 10 mg/kg prevented TBI-induced LTP impairment when compared to mice treated with tat-SCR (*p* < 0.05, 1-Way ANOVA) 7 days after TBI. Arrow indicates timing of TBS (40 pulses). **(C)** Quantification of change in fEPSP slope after 60 min following TBS normalized to baseline, set at 100%. Each point represents a hippocampal slice that was recorded with no more than two slices per animal used. **(D)** Memory impairment 7 days after TBI. Adult mice displayed memory dysfunction after contextual fear conditioning. Quantification of freezing behavior 24 h after contextual fear conditioning in a novel environment behavior was scored. (*n* = 7–22/group. *p < 0.05 compared to tat-scrambled, 1-Way ANOVA).

Given the effects of TRPM2 KO on outcomes following TBI, we tested the effect of our TRPM2 channel antagonist, tat-M2NX, administered intravenously 2 h post injury, and analyzed synaptic plasticity and memory outcomes 7 days after TBI. Figures 4B,C shows a significant impairment of LTP (117.3 ± 8.2%; *n* = 8) 7 days after TBI when compared to sham controls (167.5 ± 12.43; *n* = 5) (*p* < 0.05). tat-M2NX administered at the lowest dose (0.2 mg/kg) had no effect on LTP compared to scrambled tat-M2NX TBI group (129.9 ± 5.6%, *n* = 5; 110.4 ± 10.9%; *n* = 6, respectively; (*p* < 0.05, 1-Way ANOVA)). However, treatment with tat-M2NX at 2.0 or 10 mg/kg prevented TBI-induced LTP impairment (165.5 ± 15.7% (*n* = 8) and 186.9 ± 17.3% (*n* = 6), respectively) when compared to mice treated with tat-SCR (*p* < 0.05, 1-Way ANOVA). These results suggest that there is a dose-dependent effect of tat-M2NX on recovery of hippocampal plasticity, observed 7 days after TBI.

To investigate whether tat-M2NX had effects on learning and memory behavior 7 days following TBI, the same doses (0.2, 2.0, 10 and 20 mg/kg) were administered intravenously 2 h post-TBI. tat-M2NX administered at the lowest dose (0.2 mg/kg) had no effect on freezing behavior (Figure 4D), when compared to TBI treated with tat-SCR (22.4 ± 2.3% and 30.4 ± 3.3%; *n* = 22, respectively; *p* < 0.05, 1-Way ANOVA). However, treatment with 2.0 or 10 (47.9 ± 8.2%; *n* = 10; 52.1 ± 4.7%, *n* = 9;), respectively; prevented TBI-induced memory impairment when compared to tat-SCR (*p* < 0.05). Finally, we tested a higher dose (20 mg/kg) previously used in models of focal ischemia and observed improved memory function (62.3 ± 5.1%, *n* = 7) that is equivalent to 10 mg/kg, demonstrating maximal beneficial effect at 10 mg/kg. Together, these results demonstrate that TRPM2 channel activity plays a role in TBI-induced learning impairment and that acute administration of tat-M2NX can prevent TBI-induced learning impairments.

### Inhibiting TRPM2 receptor activity preserves hippocampal synaptic plasticity and memory function 30 days following TBI

**Figure 5:**
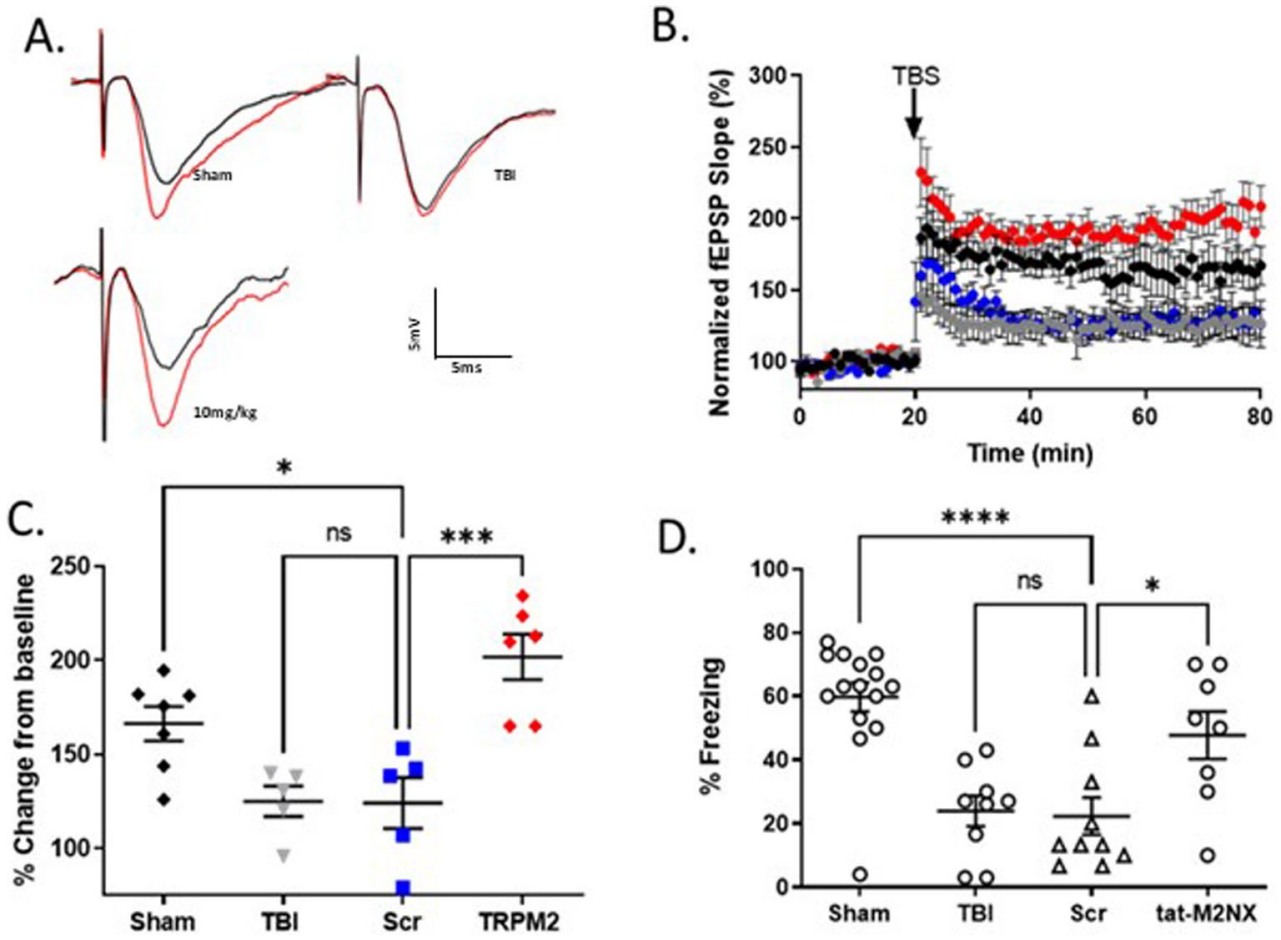
Acute taM2NX administration enhances hippocampal plasticity and memory function 30 days after recovery from TBI. **(A)** Example fEPSPs from sham-operated control mice at 30 days after injury before (black) and after (red) TBS in sch-CA1 hippocampal pathway. **(B)** Time-course of fEPSP slope from the hippocampus in sham mice (166.3 ± 9.152; *n* = 7; black filled diamonds), TBI (125.0 ± 8.061, *n* = 5; gray filled circles) and tat-scrambled treated mice in (124.4 ± 13.63, *n* = 5; blue filled squares) and tat-M2NX treated (201.8 ± 12.14, *n* = 6; red filed diamonds) 30 days after TBI. Arrow indicates timing of TBS (40 pulses). **(C)** Quantification of change in fEPSP slope after 60 min following TBS normalized to baseline, set at 100% (mean ± SEM). Each point represents a hippocampal slice that was recorded with no more than two slices per animal used. **(D)** Memory impairment 30 days after TBI. Adult mice displayed memory dysfunction after contextual fear conditioning. Quantification of freezing behavior 24 h after contextual fear conditioning in a novel environment behavior was scored. (*n* = 8–15/group. *p < 0.05 compared to tat-scrambled, 1-Way ANOVA).

We next sought to determine long-term effects of acute administration of tat-M2NX on hippocampal synaptic plasticity. Mice were administered 10 mg/kg 2 hours following TBI, with hippocampal LTP analyzed 30 days after recovery from TBI. Figures 5B,C show sham operated control mice exhibited LTP of 166.3 ± 9.2% (*n* = 7) that was significantly reduced 30d after TBI (125.0 ± 8.1%; *n* = 5), *p* < 0.05 when compared to sham controls. Mice treated with tat-Scr (10 mg/kg) 2 h after injury and tested 30d later showed no difference compared to TBI mice (124.4 ± 13.6%; *n* = 5). However, mice treated with tat-M2NX (10 mg/kg) 2 h after injury and tested 30d later showed a significant improvement of LTP (201.8 ± 12.14%; *n* = 6), indicating sustained protection of hippocampal synaptic function. To confirm sustained functional benefit, we tested memory function 30 days after TBI. Figure 5D shows that TBI-Injured mice had impaired memory function 30 days after TBI, exhibiting reduced freezing behavior, whether untreated or treated with tat-SCR (24.0 ± 4. 7%; *n* = 9 and 22.3 ± 5.8%; *n* = 10, respectively). Treatment with 10 mg/kg tat-M2NX (59.8 ± 4.6%; *n* = 15), prevented TBI-induced memory impairment when compared to tat-SCR (*p* < 0.05, 1-Way ANOVA). There were no difference in paired puse ratio or input-ouput characteristics in any of the conditions studied (Supplementary Table 2). This suggest chronic activation of TRPM2 channels impair hippocampal function and remarkably, a single dose of the novel tat-M2NX inhibitor 2 h post injury provides sustained benefit (Figure 5).

## Discussion

The current study shows that severe traumatic brain injury causes acute histological injury that results in subacute and chronic impairment of hippocampal synaptic plasticity and loss of short-term memory in a mouse model. We observed that the oxidative-stress sensitive ion channel TRPM2 contributes to acute injury in males only. The use of global TRPM2 channel knockout mice demonstrate a role for TRPM2 channels in both acute histological injury and long-term functional recovery in males. In contrast, we observed that early pharmacological inhibition of TRPM2 channels with the novel peptide inhibitor tat-M2NX failed to reduce histological injury, but did provide sustained functional benefit.

Traumatic brain injury can be classified into primary and secondary injury, albeit a somewhat oversimplification of what really represents a continuum (Werner and Engelhard, 2007). Primary injury is the immediate consequence of the physical forces on the brain, while secondary injury occurs in the hours to days after the impact and contribute to ongoing cell death and neuroinflammation (Schimmel et al., 2017; Lozano et al., 2015). The secondary injury proceeds for a few days and has been observed to exhibit ongoing injury up to 7 days after injury. However, it is during this period of histological changes that potential interventions would likely have maximal impact. Therefore, histological injury in the current study was performed 7 days after TBI, to analyze the effects of both primary and secondary injury. Secondary injury is a complex inter-related set of processes consisting of brain edema, BBB breakdown, ionic imbalances, neuroinflammation and oxidative stress which results in altered cellular function and/or cell death (Schimmel et al., 2017; Lozano et al., 2015). Unfortunately, there are currently no targeted treatments available to enhance recovery following severe TBI. Previously, we observed tat-M2NX neuroprotection in male mice following models of global and focal cerebral ischemia (Shimizu et al., 2016; Dietz et al., 2020; Cruz-Torres et al., 2020). In contrast, we did not observe reduction in histological injury following acute administration of tat-M2NX. A potential explanation for this difference is that tat-M2NX is effectively taken into neurons, with less impact on microglia and other neuro-immune pathways. Indeed, the study by Cook et al. (2010), implicated increased TRPM2 expression in microglia following rat TBI (Cook et al., 2010). It remains to be determined the specific effects of tatM2NX on various injury pathways such as neuroinflammation, edema and oxidative stress. Nonetheless, the data presented here provides evidence for improved synaptic and memory function following severe TBI, implicating TRPM2 channels in TBI-induced cognitive decline. The observation that TRPM2 KO mice exhibit reduced injury magnitude, while iv administration of tat-M2NX failed to protect raises the possibility that sustained TRPM2 channel inhibition is required. Therefore, it will be important to test repeated dosing with boosting doses of ta-M2NX. Importantly, we have strong safety data demonstrating no impact on blood pressure or heart rate when administered at the highest dose tested in the current study (Shimizu et al., 2016). Finally, it is possible that the severe TBI model utilized in the current study minimizes pharmacological inhibition and tat-M2NX would provide protection following more moderate injuries. Therefore, further studies are needed to determine the reason that acute TRPM2 inhibition failed to reduce injury.

Sex differences in incidence and outcome in traumatic brain injury have been well described. The number of male patients suffering TBI is higher than age matched females (Leitgeb et al., 2011; Peterson et al., 2019; Frost et al., 2013). Animal models have observed sex-differences in TBI outcomes, with it generally being observed that females suffer smaller injuries and less severe functional deficits than males. This has been attributed to the protective effect of female sex hormones, progesterone and estrogens. Consistent with previous studies, we observe greater injury magnitude in male animals compared to females. Interestingly, we observe significant reduction in injury volume in male TRPM2 KO mice. In contrast, we do not observe reduction in female TRPM2 KO compared to female wild-type mice. This observation is consistent with several recent studies by our group, demonstrating male-specific neuroprotection in TRPM2 KO or inhibition following models of cerebral ischemia, both focal (Jia et al., 2011; Shimizu et al., 2016; Shimizu et al., 2013) and global (Nakayama et al., 2013; Dietz et al., 2020; Shimizu et al., 2016; Dietz et al., 2021). Sex-specific response of TRPM2 channels in models of ischemic stroke has been extensively studied, with clear evidence of androgen regulation in male brain (Shimizu et al., 2013) and no impact of sex-steroid signaling in the female bran (Shimizu et al., 2016; Quillinan et al., 2014). The mechanism of male-specific activation of TRPM2 channels following traumatic brain injury remains unclear and warrants further study. Nonetheless, the current study provides strong data indicating that functional recovery and histological injury are not directly correlated and importantly, that acute inhibition of TRPM2 channels improves functional recovery.

There is increasing awareness of the interaction between acute traumatic brain injury and subsequent dementia-like symptoms (Mendez, 2017; Shively et al., 2012). Here, we demonstrate that severe traumatic injury results in sustained deficits in hippocampal synaptic plasticity and decreased short-term memory. Importantly, the magnitude of injury was chosen to result in cortical and hippocampal loss on the ipsilesional hemisphere. We did not observe any injury or neuronal loss in contralateral brain structures. Further, we observed that administration of tat-M2NX rescued TBI-induced deficits in hippocampal plasticity and memory function, despite failing to alter histological injury. This is a surprising finding that implicates a complex regulation of TRPM2 channels in the acute and chronic phase of injury. Further, these data indicate alterations in the intact contralateral hemisphere is sufficient to enhance functional recovery. Our data are consistent with an emerging perspective in the brain injury field that injury and function are not always directly correlated and that translational studies should focus on mechanisms of sustained functional benefit (Dietz et al., 2020; Dietz et al., 2021; Ng and Lee, 2019; Basak et al., 2023). Indeed, the STAIR recommendations for future development of pre-clinical translational studies recommends the use of male and female animals and neurobehavioral studies to complement histology analysis [Stroke Therapy Academic Industry Roundtable (STAIR), 1999]. We propose that the use of cellular models of learning and memory (LTP) in combination with neurobehavior provides a robust platform for translational studies and the identification of cellular mechanisms of plasticity and repair. One surprising finding of the current study is that unilateral hippocampal plasticity is sufficient to sustain memory function. Our model of severe TBI causes complete ablation of the ipsilateral hippocampus and yet, TRPM2 KO and tat-M2NX treatment enhances contralateral plasticity and memory function. This is consistent with finding in humans showing unilateral hippocampal representation of newly formed short-term memories (Burgess et al., 2002; Klur et al., 2009; Maguire and Frith, 2003).

In conclusion, our study strongly implicates TRPM2 channel activity in the acute and chronic phases of injury following severe TBI. We demonstrate that acute administration of the TRPM2 channel antagonist tat-M2NX is a promising therapeutic that provides sustained functional benefit.

## Transparency, rigor and reproducibility summary

Our translational animal experiments were conducted in accordance with the ARRIVE guidelines1. Male and female young adult (2–4 months) mice were blindly randomized to experimental or control groups, minimizing subjective bias and controlling for gender effects of TBI. Rigorous contemporaneous controls were used. Investigators administrating drug were double blinded by not knowing the animal treatment and therapeutic being administered. All analyses were performed by observers blinded to treatment allocation. All methods utilized in this study can be found in our previous publications 2-5.

## Data Availability

The raw data supporting the conclusions of this article will be made available by the authors, without undue reservation.
