# Supplementary material for: A novel peptide inhibitor of TRPM2 channels improves recovery of memory function following traumatic brain injury
Source: Front Synaptic Neurosci. 2025 Apr 25;17:1534379. doi: 10.3389/fnsyn.2025.1534379 (PMC12061953; doi:10.3389/fnsyn.2025.1534379)
Supplement: Supplementary file 1 [file Data_Sheet_1.pdf]

SUPPLEMENTAL MATERIALS:

7 Day TBI

	Sham	TBI	Scr-M2NX	0.2mg M2NX	2.0mg M2NX	10mg M2NX	-/- TBI	-/- Sham	*p* value
**Animals**	6	7	3	3	6	3	4	3	
**HC Slices**	10	12	6	5	8	6	6	6	
**PPR (pulse 2/pulse 1)**	1.48± 0.03	1.49 ± 0.04	1.42 ± 0.09	1.52 ± 0.07	1.46 ± 0.05	1.56 ± 0.03	1.49 ± 0.07	1.68 ± 0.1	0.24
**I/O (slope)**	1.80 ± 0.05	1.793 ± 0.08	1.78 ± 0.09	1.90± 0.08	1.80 ± 0.07	1.73 ± 0.06	1.85 ± 0.08	1.83 ± 0.07	0.92

Supplemental Table 1. Animals used, hippocampal (HC) slices used, paired-pulse ratios and input-output (I/O) slope obtained in sham, TBI, Scr-M2NX, .2mg, 2.0mg, 10mg, -/-TBI and -/- sham mice at 7 days post-injury. No significant differences were observed among groups in either paired-pulse ratios (p=0.24) by ANOVA or I/O slope (p = 0.92) by linear regression analysis. Data summary representative of results presented in Figures 3 and 4.

30 Day TBI

	Sham	TBI	Scr M2NX	10mg M2NX	*p* value
**Animals**	3	4	3	5	
**HC Slices**	5	7	6	5	
**PPR (pulse 2/pulse 1)**	1.47± 0.05	1.46 ± 0.08	1.48 ± 0.07	1.52 ± 0.08	0.77
**I/O (slope)**	1.81 ± 0.12	1.73 ± 0.08	1.9 ± 0.13	1.76 ± 0.09	0.81

Supplemental Table 2. Animals used, hippocampal (HC) slices used, paired-pulse ratios and input-output (I/O) slope obtained in sham, TBI, Scr-M2NX and 10mg treated mice at 30 days post injury. No significant differences observed among groups in either paired-pulse ratios (p=0.77) by ANOVA. I/O slope (p = 0.81) by linear regression analysis. Data summary representative of results presented in Figure 5.
